# Effects of *Fructus Piperis Longi *extract on fibrotic liver of gamma-irradiated rats

**DOI:** 10.1186/1749-8546-4-2

**Published:** 2009-01-30

**Authors:** Somaya Zakaria Mansour, Hanan El-Kabany

**Affiliations:** 1 Radiation Biology Department, National Centre for Radiation Research and Technology, Atomic Authority, Cairo, Egypt; 2 Health Radiation Research Department, National Centre for Radiation Research and Technology, Atomic Authority, Cairo, Egypt

## Abstract

**Background:**

A major biomarker for liver fibrosis is transglutaminase which catalyzes cross-linking of epsilon-amines and alpha-glutamyl residues among amino acids leading to fibrosis. *Fructus Piperis Longi *is a common herb used in Chinese medicine. The present study evaluates the role of the ethanol extract of *Fructus Piperis Longi *in the modulation of liver function in liver fibrosis.

**Methods:**

Plf extract (50 mg/kg) was force-fed to rats every other day 7 days before administration of thioacetamide and/or gamma irradiation. Thioacetamid 200 mg/kg was intraperitoneally administered to rats twice per week for four weeks. Rats were gamma irradiated (2 Gy/week up to a total dose of 8 Gy). Administration of Plf ext was extended during thioacetamid and/or irradiation treatment. Animals were sacrificed. Biochemical parameters in homogenised liver were tested.

**Results:**

A significant increase in transglutaminase activity and collagen content was recorded in the liver of thioacetamid-treated and/or irradiated rats. Significant increases in lipid peroxides, lipid hydroperoxides and conjugated dienes associated to significant decreases of reduced glutathione content, superoxide dismutase and catalase activities were also recorded. Administration of Plf ext treatment reduced the severity of liver fibrosis and oxidative damage which was substantiated by amelioration of liver function detected by a decrease in serum aspartate aminotransaminase, alanine aminotransferase, alkaline phosphatase, gamma glutamyltransferase activities and bilirubin (total, direct and indirect) content.

**Conclusion:**

Treatment of the ethanolic extract of *Fructus Piperis Longi *ameliorated the increase of the activity of tTG enzyme and enhanced antioxidant activities in fibrotic liver.

## Background

Fibrosis of the liver is a state of complicated end stage alteration of structure and function due to different aetiologies. Fibrosis is a consequence of different prevalent mechanisms according to the diverse causes of parenchymal damage. Fibrosis caused by chronic viral infection is initially concentrated within and around the portal tract, while fibrosis secondary to toxic/metabolic damage is located mainly in the centrolobular areas [1].

Oxidative stress, characterized by the overproduction of reactive oxygen species (ROS), which overwhelm the levels of antioxidants, has been suggested as the pathogenic factor of a number of human diseases and was reported to cause tissue damage [2]. ROS can react with cellular macromolecules such as nucleic acids, polyunsaturated fatty acids in cellular membranes and sulfhydryl bonds in proteins to cause mutagenesis, carcinogenesis and cell death.

Thioacetamide (TAA, CH_3_-C[S] NH_2_), a known fungicide used to control the decay of fruits [3] was shown to be S-oxidized at the thioamide group to TAA sulfoxide (CH_3_-C[SO] NH_2_) and subsequently di-Soxide (CH_3_-C[SO_2_] NH_2_) in the liver. The reactive intermediates in this pathway covalently bind to hepatic macromolecules and eventually cause liver injury [4,5], whereby free radical-mediated lipid peroxidation contributes to the development of TAA induced liver fibrosis [6,7]. Prolonged administration of TAA causes hyperplastic liver nodules, liver cell adenomas and hepatocarcinomas. The free radicals produced during TAA metabolism interfere with ribosomal activity, thereby hindering protein synthesis [8]. The biochemical and morphological changes observed in TAA-induced rat liver injury resemble to a large extent human liver disease and could serve as a suitable model for studying the causes of human liver fibrosis and cirrhosis [9].

Tissue fibrosis is associated with the increase of the tTG activity and accumulation of ECM [10]. In liver fibrosis induced in rats by carbon tetrachloride (CCl_4_) and in human patients with an acute liver disease, Mirza *et al*. [11] found a dramatic rise in tissue transglutaminases (tTG) activity. The enzyme catalyzes the specific cross-linking of ε-amines and α-glutamyl residues among amino acids [12]. This activity leads to the cross-linking of extracellular matrix (ECM) proteins thereby increasing the deposition [13] of such proteins and their resistance to proteolytic enzymes, which leads to tissue fibrosis [14,15]. Several studies specifically described the role of tTG in cross-linking of fibronectin, osteonectin, osteopontin, laminin and other extracellular matrix components [12].

The pathogenesis of liver fibrosis is not clear; however, it was suggested that an increase of ROS coupled with a decrease in body antioxidant system activity play an important role in the pathological changes, particularly in the cases of radiation exposure and liver toxicity [16]. Radiation exposure may cause disruption of normal cell membranes as a result of direct interaction of radiation with cellular membranes or through the action of free radicals produced by radiation [17].

Several endogenous protective mechanisms may limit ROS and the damage caused by them [18]. However, this protection may be insufficient. When the formation of ROS is excessive, additional protective mechanisms of dietary, antioxidants may help maintain liver functions. Several natural antioxidants were proposed to prevent and treat hepatopathies induced by oxidative stress [19]. Rich in flavonoids, *Fructus Piperis Longi *(*Bibo*, long pepper) is used in Chinese medicine to treat various conditions such as jaundice and allergy [20]. It has been demonstrated to be anti-tussive, anti-asthmatic, anti-allergic, anti-tubercular, antipyretic, hypotensive, hypoglycemic, antihelmentic and coronary vasodilatory [21]. The aim of the present study is to investigate the hepatoprotective activity of *Fructus Piperis Longi *against liver fibrosis.

## Methods

### Materials and instrument

*Fructus Piperis Longi *was obtained from the local market. All chemicals and reagents used in the experiment were of analytical grade and purchased from either Merck (Germany) or Sigma Aldrich Chemie (Germany). Assay kits for testing alkaline phosphatase (ALP), alanine aminotransferase (ALT), aspartate aminotransaminase (AST), gamma glutamyltransferase (GGT), bilirubin and total protein were supplied by Diamond Diagnostics (Egypt).

Instrument included electric digital balance (Shimadzu, Type Ay 220, Japan), pH meter (Jenway, UK), homogenizer (Glas-col, TERRE HAUT, USA), cooling centrifuge (Memmert, Model K23, Germany), centrifuge (Janetzki, Model T30, Germany), shaker incubator (Lab-Line Instruments, USA) and spectrophotometer (Helios, UV/Visible, UK).

### Experimental animals

All animal treatment procedures conformed to the National Institutes of Health (NIH) guidelines [22]. Sprague Dawley male albino rats (170–220 g) were used in this study. Animals were obtained from the National Centre for Radiation Research and Technology (NCCRT), Cairo, Egypt. The animals were housed in cages and maintained under standard conditions of ventilation, temperature and humidity. Animals received standard food pellets and water *ad libitum*.

### Gamma irradiation procedure

Irradiation of animals was carried out at the National Centre for Radiation Research and Technology (NCRRT) in Cairo, Egypt, with a Gamma cell-40 (Cesium-137 irradiation units, Canada). The irradiation dose rate was 0.61 Gy/min. Animals (whole body) were exposed to 2 Gy per week at a total dose of 8 Gy, one day after TAA administration.

### Induction of liver fibrosis

Liver fibrosis was induced by intraperitoneal administration of 200 mg/kg TAA twice per week for four weeks according to El Borai *et al*. [23].

### Preparation of Fructus Piperis Longi extract

The ethanolic extract of *Fructus Piperis Longi *was prepared according to Christina *et al*. [21]. *Fructus Piperis Longi *was obtained from the local market and was dried and powdered. About 500 g of dry powder was extracted with 5L of ethanol at 60–70°C for 72 hours by continuous hot percolation with a Soxhlet apparatus. The ethanolic extract was then filtered and concentrated by vacuum distillation to dry. The yield for 500 g was 37 g. This dried extract was then stored at 4°C until use. Rats were force-fed 50 mg/kg of distilled water per day for five weeks starting from seven days before TAA administration.

### Animal groups

The experimental animals were divided into eight groups (n = 6), namely (1) Control: healthy animals received distilled water; (2) Plf ext: animals received *Fructus Piperis Longi *extract; (3) TAA: animals were injected with TAA; (4) Plf ext + TAA: animals received *Fructus Piperis Longi *extract and were injected with TAA; (5) γ irradiation: animals were exposed to γ irradiation; (6) Plf ext + γ irradiation: animals received *Fructus Piperis Longi *extract and were exposed to γ irradiation; (7) TAA + γ irradiation: animals were injected with TAA and exposed to γ irradiation and (8) Plf ext + TAA + γ irradiation: animals received *Fructus Piperis Longi *extract and were injected with TAA and exposed to γ irradiation.

Rats of all groups received the last irradiation exposure on the day before overnight fasting and sacrifice. Blood samples were collected by heart puncture. Plasma of each blood sample was separated and kept frozen for biochemical assays. Liver samples were kept at -80°C until biochemical assays. Liver tissue homogenate (10% w/v) in phosphate-buffered-saline (0.02 M sodium phosphate buffer with 0.15 M sodium chloride, pH7.4) was prepared with a portion of liver homogenized in a glass tissue homogenizer with a Teflon pestle.

### Biochemical assays

Tissue transglutaminase (tTG) activity in 100 μl liver homogenate was determined according to the direct spectrophotometric method by De Macedo *et al*. [24]. Total protein content in liver tissue was determined according to the method by Henry [25] to calculate the specific enzyme activity of tTG in the liver. Liver collagen content was determined according to Woessner [26].

Reduced glutathione concentration (GSH) in liver was determined according to Beutler *et al*. [27]. Superoxide dismutase (SOD) activity in liver was measured according to Minami and Yoshikawa [28]. The colorimetric assay for liver catalase activity (Cat) was carried out according to Sinha [29]. Lipid peroxides (LP) indicated by the formation of malondialdehyde (MDA) was assessed in liver homogenates according to Yoshioka *et al*. [30]. Lipid hydroperoxides (LHP) in liver was determined according to the Fox method described by Jiang *et al*. [31]. The levels of conjugated dienes (CD) in liver were measured according to Rechnagel and Gglende and Nowak *et al*. [32,33].

Plasma ALP activity was determined according to Teitz [34]. Activities of ALT and AST were determined colorimetrically according to Reitman and Frankel [35]. Plasma gamma GGT activity was measured kinetically according to Szasz [36]. Plasma bilirubin (total, direct and indirect) contents were determined according to Perry *et al*. [37].

### Statistical analysis

The SPSS (version 10) was used in data analysis. Data were analyzed with one-way analysis of variance (ANOVA) followed by a *post hoc *test (LSD alpha) for multiple comparisons. The data were expressed as mean ± standard deviation (SD). P values < 0.05 were considered to be statistically significant.

## Results

Administration of *Fructus Piperis Longi *ethanol extract (Plf ext) to rats, by force-feeding, for a period of five weeks, did not show significant changes in all the studied parameters, indicating that the extract did not affect the liver functions (Tables 1, 2, 3, 4, 5).

**Table 1:** Transglutaminase (tTG) activity and collagen content in liver tissue homogenates of rats under different treatment conditions

**Groups**	**tTG (anilide/umol/mg protein/min)**	**Collagen (mg/g wet tissue)**
**Control**	1.26 ± 0.182	3.96 ± 0.346
**Plf ext**	1.32 ± 0.111	4.03 ± 0.221
**TAA**	2.87 ± 0.166	6.14 ± 0.318
**a**	P = 0.0001	P = 0.0001
**Plf ext+TAA**	1.58 ± 0.085	4.60 ± 0.163
**a**	P = 0.007	P = 0.013
**b**	P = 0.0001	P = 0.0001
**γ radiation**	2.26 ± 0.070	4.56 ± 0.253
**a**	P = 0.0001	P = 0.019
**Plf ext+γ radiation**	1.37 ± 0.082	4.20 ± 0.499
**a**	NS	NS
**c**	P = 0.0001	NS
**TAA+γ radiation**	3.49 ± 0.185	6.77 ± 0.075
**a**	P = 0.0001	P = 0.0001
**b**	P = 0.0001	P = 0.015
**c**	P = 0.0001	P = 0.0001
**Plf ext+TAA+γ radiation**	1.76 ± 0.065	5.13 ± 0.125
**a**	P = 0.0001	P = 0.0001
**b**	P = 0.0001	P = 0.0001
**c**	P = 0.0001	P = 0.023
**d**	P = 0.0001	P = 0.0001

Each value represents mean ± SD of 6 determinations.

a: significance over control group

b: significance over TAA group

c: significance over γ radiation group

d: significance over TAA+γ radiation group

NS: no significance

**Table 2:** SOD and Cat activities and GSH content in liver tissue homogenates of rats under different treatment conditions

**Groups**	**SOD (μg/g wet tissue)**	**Cat (μmol/g wet tissue)**	**GSH (mg/g wet tissue)**
**Control**	12.06 ± 0.701	119.36 ± 9.799	24.75 ± 1.035
**Plf ext**	12.38 ± 0.499	119.37 ± 5.334	25.06 ± 0.752
**TAA**	10.79 ± 0.054	101.40 ± 3.561	21.02 ± 0.699
**a**	P = 0.0001	P = 0.0001	P = 0.0001
**Plf ext+TAA**	11.84 ± 0.398	118.89 ± 4.522	23.26 ± 0.586
**a**	NS	NS	P = 0.0001
**b**	NS	P = 0.001	P = 0.0001
**γ radiation**	11.52 ± 0.151	99.63 ± 9.279	20.60 ± 0.793
**a**	NS	P = 0.0001	P = 0.0001
**Plf ext+γ radiation**	11.88 ± 0.031	117.32 ± 9.122	23.11 ± 0.867
**a**	NS	NS	P = 0.0001
**c**	NS	P = 0.001	P = 0.0001
**TAA+γ radiation**	10.64 ± 0.316	91.01 ± 6.793	19.86 ± 0.263
**a**	P = 0.0001	P = 0.0001	P = 0.0001
**b**	NS	P = 0.041	P = 0.009
**c**	P = 0.002	NS	NS
**Plf ext+TAA+γ radiation**	11.82 ± 0.880	111.82 ± 14.453	22.80 ± 0.594
**a**	P = 0.001	NS	P = 0.0001
**b**	P = 0.001	P = 0.041	P = 0.0001
**c**	NS	P = 0.018	P = 0.0001
**d**	P = 0.0001	P = 0.0001	P = 0.0001

Each value represents mean ± SD of 6 determinations.

a: significance over control group

b: significance over TAA group

c: significance over γ radiation group

d: significance over TAA+γ radiation group

NS: no significance

**Table 3:** Lipid peroxides (LP), lipid hydroperoxide (LHP) and conjugated diene (CD) in liver tissue homogenates of rats under different treatment conditions

**Groups**	**LP (μg/g tissue)**	**LHP (μM)**	**CD (Abs_234_/g tissue)**
**Control**	108.27 ± 7.246	17.81 ± 0.841	2.20 ± 0.091
**Plf ext**	114.72 ± 5.170	17.90 ± 0.759	2.20 ± 0.068
**TAA**	169.04 ± 16.914	21.64 ± 1.995	3.14 ± 0.042
**a**	P = 0.0001	P = 0.003	P = 0.0001
**Plf ext+TAA**	127.47 ± 2.480	17.79 ± 2.949	2.30 ± 0.200
**a**	P = 0.003	NS	NS
**b**	P = 0.0001	P = 0.003	P = 0.0001
**γ radiation**	135.54 ± 13.191	20.91 ± 0.833	2.54 ± 0.191
**a**	P = 0.0001	P = 0.008	P = 0.002
**Plf ext+γ radiation**	123.98 ± 4.606	17.52 ± 2.553	2.24 ± 0.043
**a**	P = 0.012	NS	NS
**c**	NS	P = 0.008	P = 0.006
**TAA+γ radiation**	182.93 ± 9.560	21.92 ± 1.958	3.20 ± 0.015
**a**	P = 0.0001	P = 0.001	P = 0.0001
**b**	P = 0.025	NS	NS
**c**	P = 0.0001	NS	P = 0.0001
**Plf ext+TAA+γ radiation**	142.04 ± 14.092	17.88 ± 2.247	2.47 ± 0.116
**a**	P = 0.0001	NS	P = 0.011
**b**	P = 0.0001	P = 0.003	P = 0.0001
**c**	NS	P = 0.01	NS
**d**	P = 0.0001	P = 0.001	P = 0.0001

Each value represents mean ± SD of 6 determinations.

a: significance over control group

b: significance over TAA group

c: significance over γ radiation group

d: significance over TAA+γ radiation group

NS: no significance

**Table 4:** ALT, AST, ALP and GGT activities in plasma of rats under different treatment conditions

**Groups**	**ALT (U/l)**	**AST (U/l)**	**ALP (U/l)**	**GGT (U/I)**
**Control**	15.54 ± 1.987	8.00 ± 0.503	50.11 ± 1.241	1.00 ± 0.00
**Plf ext**	15.44 ± 2.001	8.18 ± 0.804	49.36 ± 1.017	1.08 ± 0.144
**TAA**	23.58 ± 1.282	16.04 ± 3.380	94.35 ± 2.431	4.66 ± 0.212
**a**	P = 0.0001	P = 0.0001	P = 0.0001	P = 0.0001
**Plf ext+TAA**	17.03 ± 1.92	9.29 ± 0.419	54.24 ± 1.417	2.30 ± 0.282
**a**	NS	NS	P = 0.0001	P = 0.0001
**b**	P = 0.0001	P = 0.0001	P = 0.0001	P = 0.0001
**γ radiation**	19.91 ± 1.371	14.37 ± 0.567	88.00 ± 1.763	2.62 ± 0.247
**a**	P = 0.0001	P = 0.0001	P = 0.0001	P = 0.0001
**Plf ext+γ radiation**	17.17 ± 1.899	9.26 ± 0.585	51.98 ± 1.592	1.43 ± 0.087
**a**	NS	NS	NS	P = 0.036
**c**	P = 0.006	P = 0.0001	P = 0.0001	P = 0.0001
**TAA+γ radiation**	24.60 ± 1.268	21.20 ± 0.560	104.40 ± 3.241	5.78 ± 0.358
**a**	P = 0.0001	P = 0.0001	P = 0.0001	P = 0.0001
**b**	NS	P = 0.0001	P = 0.0001	P = 0.0001
**c**	P = 0.0001	P = 0.0001	P = 0.0001	P = 0.0001
**Plf ext+TAA+γ radiation**	19.85 ± 0.813	12.20 ± 1.344	55.99 ± 2.794	2.71 ± 0.276
**a**	P = 0.0001	P = 0.0001	P = 0.0001	P = 0.0001
**b**	P = 0.0001	P = 0.0001	P = 0.0001	P = 0.0001
**c**	NS	P = 0.011	P = 0.0001	NS
**d**	P = 0.0001	P = 0.0001	P = 0.0001	P = 0.0001

Each value represents mean ± SD of 6 determinations.

a: significance over control group

b: significance over TAA group

c: significance over γ radiation group

d: significance over TAA+γ radiation group

NS: no significance

**Table 5:** Bilirubin total, direct and indirect concentration in plasma of rats under different treatment conditions

**Groups**	**Bilirubin total (mg/ml)**	**Bilirubin direct (mg/ml)**	**Bilirubin indirect (mg/ml)**
**Control**	0.923 ± 0.022	0.146 ± 0.006	0.777 ± 0.026
**Plf ext**.	0.867 ± 0.060	0.145 ± 0.007	0.722 ± 0. 061
**TAA**	2.280 ± 0.169	0.250 ± 0.024	2.031 ± 0.166
**a**	P = 0.0001	P = 0.0001	P = 0.0001
**Plf ext+TAA**	1.015 ± 0.033	0.184 ± 0.006	0.832 ± 0.028
**a**	P = 0.049	P = 0.0001	NS
**b**	P = 0.0001	P = 0.0001	P = 0.0001
**γ radiation**	1.307 ± 0.071	0.199 ± 0.014	1.103 ± 0.074
**a**	P = 0.0001	P = 0.0001	P = 0.0001
**Plf ext+γ radiation**	0.923 ± 0.033	0.162 ± 0.009	0.761 ± 0.030
**a**	NS	P = 0.020	NS
**c**	P = 0.0001	P = 0.0001	P = 0.0001
**TAA+γ radiation**	3.137 ± 0.076	0.281 ± 0.008	2.856 ± 0.079
**a**	P = 0.0001	P = 0.0001	P = 0.0001
**b**	P = 0.0001	P = 0.0001	P = 0.0001
**c**	P = 0.0001	P = 0.0001	P = 0.0001
**Plf ext+TAA+γ radiation**	1.310 ± 0.063	0.226 ± 0.005	1.084 ± 0.062
**a**	P = 0.0001	P = 0.0001	P = 0.0001
**b**	P = 0.0001	P = 0.0001	P = 0.0001
**c**	NS	P = 0.0001	NS
**d**	P = 0.0001	P = 0.0001	P = 0.009

Each value represents mean ± SD of 6 determinations.

a: significance over control group

b: significance over TAA group

c: significance over γ radiation group

d: significance over TAA+γ radiation group

NS: no significance

As shown in Table 1, TAA significantly increased (P = 0.0001) liver collagen content and tTG activity. Irradiated rats showed significantly increased liver collagen content (P = 0.019) and tTG activity (P = 0.0001). In the TAA + irradiation group, significant increases (P = 0.0001) in liver collagen content and tTG activity were observed. Treatment of Plf ext significantly ameliorated (P = 0.0001, no significance, 0.0001, 0.0001, 0.0001 and 0.0001 respectively) the increase of collagen content and tTG activities in the rats that received TAA or γ-irradiation or both (Table 1).

TAA induced significant decreases (P = 0.0001) in liver GSH content, SOD and Cat activities (Table 2), which were parallel to significant increases in LP (P = 0.0001), LPH (P = 0.003) and CD (P = 0.0001) content (Table 3). Irradiated rats showed significant decreases (P = 0.0001) in liver GSH content and Cat activity in association with significant increases in LP (P = 0.0001), LPH (P = 0.008) and CD (P = 0.002) content (Tables 2 and 3). In the TAA + irradiation group, significant decreases (P = 0.0001) in liver GSH content, SOD and Cat activities in association with significant increases in LP (P = 0.0001), LPH (P = 0.001) and CD (P = 0.0001) content were observed. Treatment of Plf ext significantly reduced (P = 0.0001) oxidative stress in the rats that received TAA or γ-irradiation or both.

Significant increases (P = 0.0001) of plasma ALT, AST, ALP and GGT activities were observed in rats that received TAA or γ-irradiation or both. Treatment of Plf ext ameliorated (P = 0.0001) these increases (Table 4).

Significant increases (P = 0.0001) in the content of total, direct and indirect bilirubin were observed in the rats that received TAA or γ-irradiation or both. Treatment of Plf ext ameliorated (P = 0.0001) these increases (Table 5).

**Figure 1:**
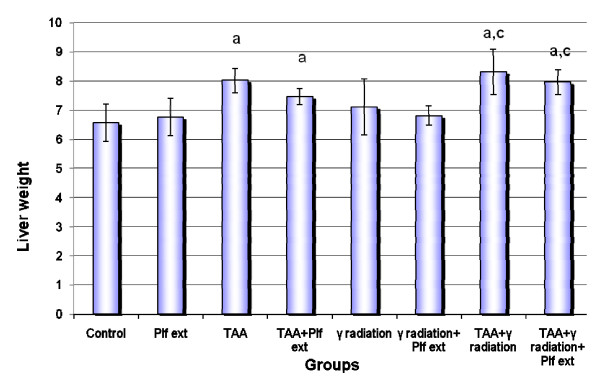
**Rats' liver weight under various treatment conditions**. Each value represents the mean ± SD of 6 determinations. a: significance of treatment over control P = 0.0001, 0.021, 0.0001 and 0.001 respectively. c: significance of treatment over γ irradiation group P = 0.003 and 0.030 respectively.

Figure 1 shows a significant increase in the liver weight of the rats that received TAA or γ-irradiation or both. Administration of Plf ext did not significantly ameliorate liver weight.

## Discussion

In the present study, parameters of liver fibrosis induced by TAA with or without radiation exposure were shown as an increase of liver weight, significant increases in liver tTG activity and collagen content associated with significant decreases in GSH content, SOD and Cat activities and increases in LP, LHP and CD content. Hepatic damage was indicated by significant increases of serum AST, ALT, ALP activities and bilirubin content.

The increase in tTG activity may be attributed to the increased binding of the nuclear factor-kappaB (NF-κB) to the NF-κB motif of the tTG promoter, where tTG gene expression increases during hepatic injury and fibrosis [38]. The concomitant increase of both hepatic collagen and tTG activity may be explained by the dual effect exerted by the NF-κB, which is induced by oxidative stress [39]. Nevertheless, the association between tTG activity and fibrosis may involve other factors such as the factor-beta (TGF-β), major fibrogenic growth factors, where tTG activates the latent TGF-μ1, which in turn leads to *de novo *synthesis of tTG [40]. The increase of tTG activity may also be a consequence of GSH depletion and mitochondrial dysfunction [41].

The depletion in GSH content may be due to the oxidation of sulfhydryl group and diminished activity of glutathione reductase [42]. The significant decrease in the activity of antioxidant enzymes may be caused by cell membrane damage and alterations in dynamic permeability of membranes due to peroxidation, followed by the release of intracellular enzymes to the blood stream [43]. In addition, an excess of ^•^OH causes oxidative damage to enzymes, resulting in the modification of their activities [43,44]. The marked increase in MDA levels is likely to be a result of the inactivation of scavenger enzymes induced by ROS [44-46]. According to Ueda *et al*. [47], the generation of lipid peroxide and its appearance in the animal's liver may be a result of a chain of reactions or may be initiated by an indirect mechanism that enables the escape from anti-oxidation.

*Fructus Piperis Longi *has recently been proposed as a chemopreventive agent for its antioxidant activities [48,49]. The present study showed that treatment of Plf ext significantly reduced liver fibrosis as evidenced by significant decreases of tTG activity and collagen content, with concomitant enhancement of the antioxidant status and improvement of liver functions. The results are consistent with other reports on the role of polyphenols against oxidative stress [39] because Plf ext is rich in polyphenols [50] which may up-regulate the antioxidant [51-53], thereby decreasing the free radical-induced lipid peroxidation [51].

The increased resistance of liver tissue against liver fibrosis and oxidative stress after treatment was shown by the significant decreases in serum ALT, ALP and AST activities and bilirubin content. The results are consistent with a previous study which demonstrated that the ethanolic extract of *Fructus Piperis Longi *possessed hepatoprotective activity lowering serum enzymes ALT and AST [54]. Further studies NF-κB, tTG gene expression and TGF-B would help elucidate the mechanism of action

## Conclusion

Treatment of the ethanolic extract of *Fructus Piperis Longi *ameliorated the increase of the activity of tTG enzyme and enhanced the antioxidant activities in fibrotic liver.

## Abbreviations

tTG: transglutaminase; Plf ext: *Fructus Piperis Longi *ethanol extract; TAA: thioacetamide; LP: lipid peroxides; LHP: lipid hydroperoxides; CD: conjugated dienes; GSH: reduced glutathione; SOD: superoxide dismutase; Cat: catalase; AST: aspartateaminotransaminase; ALT: alanine aminotransferase; ALP: alkaline phosphatase; GGT: gamma-glutamyltransferase; ROS: reactive oxygen species; SD: standard deviation; NF-κB: nuclear factor-kappaB; TGF-β: tumour growth factor-beta; Abs_234_: Absorbance at 234 nm.

## Competing interests

The authors declare that they have no competing interests.

## Authors' contributions

SZM designed the study, supervised the experiments, prepared Plf ext and wrote the manuscript. HEK performed the animal experiments. Both authors supervised the research assistants to carry out clinical chemistry assays. Both authors read and approved the final manuscript.
